# Experiments in Inoculation, and Pathological Researches and Natural History to Develop Helminthiasis as Contagion

**Published:** 1845-10

**Authors:** 


### ART. IX.

*Impversuche und natur-historisch pathologische Untersuchungen zur Erforschung der Helminthiasis als Contagium.* Einer Inauguralschrift von P. F. H. KLENCKE.

*Experiments in Inoculation and Pathological Researches and Natural History to develop Helminthiasis as Contagion.* An Inaugural Dissertation by P. F. H. KLENCKE.

THE old doctrine of the parasitic origin of certain diseases has received a new development since the microscope has become more generally applied to pathology. There is nothing in the doctrine itself which should lead the physician to reject it without inquiring into the facts on which it is based; and the inquiry itself is comparatively easy because (as in the case before us) it resolves itself mainly into a judgment as to the truthfulness of the narrator and to his competence to observe facts and describe them accurately. The researches of Henle, Langenbeck, jun. and others, on the parasitic origin of certain diseases may now indeed be considered amongst the established contributions to pathology.

In the work now under judgment, we have the inquiry as to the parasitic origin of disease limited to researches into the natural history of certain entozoa. The author makes little or no reference to the pathology of cells or of microscopic mass-like organisms. It is simply an inquiry into the generation, development, and habits of the parasitic animals found within the human body, together with observations on the pathological phenomena they excite, and the therapeutic means necessary for their removal.

Dr. Klencke divides human entozoa into two classes, according as their habitation is within the intestinal canal or without it. In the latter class are the various kinds of hydatids, the trichina spiralis and the distoma hepaticum; in the former, ascarides, bothryocephali and tænia solium.

*Hydatids.* These may be divided into five orders: 1, hydatis spuria; 2, acephalocystis; 3, echinococcus; 4, polycephalus or cænurus; 5, cysticercus. The cyst is the common characteristic of these animals, in which vesicular structures may be discovered with the naked eye. Dr. Klencke includes in the term hydatid all those bladder-like structures found in the living organized tissues, which either possess the faculty of motion or of propagation. According to this definition the hydatid is to be considered as an individual.

*Hydatis spuria.* This is often mistaken for a true hydatid. According to Klencke it is a cellula primordialis hydropica subindividuata. He has often detected it in the brain and spinal cord, and seen it in every stage of development. Its formation thus happens: the membrane of a primordial cell has an increased power of endosmosis and a diminished power of assimilation. Fluids are thus imbibed, while no granular matter is deposited. All the plastic activity of the enlarged cell that is not directed to assimilation is wasted after the early disappearance of the nucleus in the production of brood-cells which take on an action like that of the mother-cell, and bursting the latter, assume an independent existence. Their origin is in fact analogous to that of carcinoma.

*Acephalocystis.* This is not an abnormal primordial cell as the preceding, but a true animal. It is a rare species, but is often confounded with the hydatis spuria, and other parasites. Klencke met with it only thrice in twenty-one cases presented to him as examples of the disease. It is found of various magnitudes, but is usually about the size of a lentil or a pea, and is of an opal colour, particularly when found in the brain. The vesicle consists of two membranes: the one like an epithelium, fine and shining, and having a cellular organization; the other porous, tense, yellowish, and sometimes fibrous. Occasionally little canal-like, membranous projections pass through the pores to the cavity within. The latter contains a clear fluid in which the fibrinous canals float; these canals being probably intestinal openings. The cavity also contains a central body of about $\frac{1}{12}$—$\frac{1}{2}$ line in magnitude, and presents a caseous coagulated appearance. This body consists of microscopic cells, which are in fact the ovaries of the animal. Sometimes the ovary is hard and shining not unlike ivory; this condition Klencke considers to be a pathological state. The relations of the acephalocyst hydatid to the next species are curious; it is simply a mass of the ova of the

*Echinococcus.* In form this hydatid is somewhat like a pear, and is inclosed in a vesicle containing a clear yellowish fluid. If the general cyst (made up of cellular tissue) be opened, it is found to contain a number of these smooth elastic vesicles. Part are adherent to the inner surface, part swim about freely within the cavity. The upper portion of the creature consists of a flat surface set round with a circlet of little arms or hooks which it can elevate, depress, and direct at pleasure. They are hollow, and are made up of longitudinal and circular fibres. At that part of the body which may be termed the neck, there are sometimes two, sometimes four little appendages which look like a suction apparatus. These are only observed in adults. The ovaria are situate beneath the inner surface of the epithelium. According to Klencke's researches, the parent dies when the ova have advanced in development to a certain stage, and the latter then constitute an acephalocyst.

*Polycephalus cœnurus.* This curious hydatid consists of an irregular shaped vesicle to which are attached a number of polypus-like necks, each of which is a distinct animal, and which vary in size according to their age and stage of development, thus presenting an opportunity for examining each stage. Some may be observed to be perfectly developed while others are in the embryo state, and appear like little holes or dimples in the cyst as if it had been pressed inwards at this point from without.

The perfect animal consists of a retractile neck appended to a fluid sac or cæcum, which appears to be formed by the doubling in of the membrane of the common cyst. This latter consists of two layers,—an external and fibrous, and an inner or epithelial. The latter runs to form the cæcum or blind sac of the animal; from the former is developed the neck or so-called head. The head has appended to it a sheath surrounded with a circlet-like formation of long and narrow arms, sometimes twenty in number. These hydatids, like polypi, multiply by buds, or separations from the parent sac.

5. *Cysticercus.* This animal is conical in form, and consists of a neck and appendent vesicle. It is generally independent of any general cyst, and whenever the latter is found in connexion with the creature, it may be considered as the rudiment of the parent animal, or as a product of exudation and of new formation out of the surrounding tissue. The vesicle of the cysticercus consists of two membranes, an inner and an outer. The outer membrane contains transverse and circular fibres, developed at the neck into a true muscular structure by which that appendage is moved. The inner or epithelial membrane has a cellular character. The upper part of the neck terminates in a conical point whose upper end is surrounded by a double circle of long and short arms or rays, often more than thirty in number, which are capable of being elevated or depressed at will. On the side of this cone are four hollow half spheres in the centre of which is an opening furnished with a sphincter.

The cyst or vesicle of adult cysticerci contains a number of regularly formed small bodies which are made up of a transparent structure resembling the vitreous humour, and which Klencke considers to be ova. These bodies have also been found externally to the cyst. The reproduction of the cysticercus takes place by the collapse or destruction of the mother-cyst, so soon as these ova have attained a certain degree of development.

*Pathological relations of hydatids.* We shall now follow our author through his researches into the morbid phenomena induced by the presence of hydatids, and into the special organs in which they are developed.

*Hydatids of the brain.* All the preceding kinds of hydatids are found in the brain, but the symptoms differ according to the seat and habits of the hydatid. The hydatis spuria, however, is that most frequently observed, but since it is more nearly allied to the elementary constitution of the brain than the true animal hydatids, being in fact an imperfect or abnormal cell reproducing itself by blastidia, the morbid phenomena it induces are not strongly marked, and it may indeed exist in the brain without exciting any indications of its presence whatever. Klencke has seen instances in which the fibrils of nerves were compressed, and the neuroma itself separated and destroyed by interstitial growths of a vascular, cellular, or fibrous nature, and yet no indications of these changes were to be observed during life. The false hydatid is found in almost every part of the brain. Klencke has often noticed them in the fourth ventricle, in the fornix, the substance of the hemispheres, the optic nerves and commissure, the pons, between the surface of the brain and the arachnoid, and between the latter and the dura mater. In one case they were the immediate cause of amaurosis; in another they occupied nearly the whole of both hemispheres inducing anæsthesia, dullness of intellect, and loss of memory. The pain they cause is constant, and in this they differ from true hydatids, the pain of the latter being intermittent. The hydatis spuria is seldom inclosed in a general cyst, but is scattered throughout the part affected, in groups of different magnitudes, and causes no change in the surrounding cerebral substance, except that which would follow on compression.

The acephalocysts are often confounded with the preceding. They are quite distinct, and are simply the ovaria of the echinococci. They are generally combined within a cyst, with which, however, they have no anatomical connexion, and which has a true serous character, containing serum, and having a capillary system. Klencke has seen them in the substance of the hemispheres, in the lateral ventricles, and in the choroid plexus.

The symptoms of acephalocyst hydatids vary much. Generally there is headache, but not more intense than is usual in other diseases. In plethoric subjects, apoplexy may be easily induced as a consequence of their irritating action on the vascular system. Peculiar affections of the muscular system, ending ultimately in epilepsy, result from their development in the pons, fornix, crura cerebri, hemispheres of the cerebellum, or base of the medulla oblongata, or when they compress those structures. When limited to the cerebral hemispheres, they induce only stupor, or headache, and in extreme cases, apoplexy; and if the hydatids be numerous, the respiration is slower. If seated in the corpus callosum the pulse is rendered slow, but quickened if they be in the pes hippocampi. Acephalocysts in the corpora striata, thalamus, and crura cerebri are indicated by hallucinations, flashes of light, blindness, spasmodic colic, and spasm of the stomach; if they be in the corpora quadrigemina, there is paralysis of the colon, and of the intestinal canal and bladder, with abolition of the sexual function if the cerebellum be implicated. These statements are apparently made by the author as the result of observation, (one very interesting case is fully detailed,) but he seems to doubt how far they should be depended upon in diagnosis without further confirmation.

The *polycephalus* excites the rotatory disease in sheep, and has been observed in man to induce headache, rotation, loss of memory, and insensibility to light. The third ventricle and the aqueduct of Sylvius are their most usual locality, but they have been seen in the substance of the pons and in the lateral ventricles.

The cysticercus is commonly found in the choroid plexus. Whenever Klencke has found it here, he has also detected it in other organs and structures of the body.

The periodic headache and vertigo characteristic of the two last-mentioned species are dependent, Klencke thinks, upon the greater or less movement of the animal. Every motion of the neck on the crown of hooks around it must constitute an irritation equal to the sudden production of these phenomena. Inflammation of the brain, the formation of serous sacs, and serous effusion are the usual concomitants of cerebral hydatids.

*Case of hydatids of the brain.* We record this case as an interesting contribution to cerebral pathology. The patient was a man, aged 40, a gourmand, and addicted to sexual intercourse. For some years he had suffered from a profuse gonorrhœa, when he gradually became hypochondriacal. In addition to a very peculiar expression of countenance, his pupils were dilated, and his eyelids were continually opening and shutting spasmodically. He also experienced slight convulsive twitches, and pain in the course of the trigeminus and its branches which were much increased if he spoke or read much. Subsequently the patient complained of a trembling of the hand when he attempted to write; and when he closed his eyes or thought in the dark he experienced a sudden gush of tears. There was a remarkable sympathy between the state of his eyes and his stomach; when the expression of his face was the most heavy and dull, his pupil most dilated, and the appearance of his eye most glossy, he was proportionately affected with bulimia. He was at last suddenly attacked by paroxysms of vertigo, intolerance of light, difficulty in swallowing and speaking, so that he stuttered; and about every second day had apoplectic convulsions. All these symptoms remitted in a remarkable manner after (with the advice of a friend) drinking in despair a quantity of brandy. This circumstance confirmed Klencke in the opinion he had already formed as to the presence of polycephali or cysticerci in the brain, for he had learnt, both practically and by experiment, that alcohol acted as a poison to these two species of hydatids, and changed them into a hard torpid mass.

As, however, the symptoms again returned, the nitrate of silver was administered in doses of gr. $\frac{1}{8}$ with manifest advantage. The bulimia was relieved together with the tremor of the hands, the convulsive action of the eyelids was also removed, and the expression of the countenance improved. The remedy was discontinued in about a month as the skin began to be discoloured.

Our author then lost sight of the patient for two months. During that time various remedies which we need not enumerate were administered. On again seeing him Klencke found his countenance sunken and distorted, and the sense of smell in the right nostril lost; he stuttered much, the limbs trembled yet were not paralysed, the tongue was tremulous and drawn to the left, and his digestive powers were at a minimum. He died suddenly in convulsions.

*Post-mortem appearances* forty-eight hours after death. A large mass of hydatids consisting partly of acephalocysts, and partly of false hydatids were found between the pia mater and arachnoid; along the falx cerebri there was a mass of them as large as a cocoa-nut. The pia mater in the ventricles and choroid plexus was covered with numerous hydatid masses, the third ventricle being filled with them. The right foramen was distended to the size of a kidney-bean by a number of acephalocysts attached to organized fibrine; the right corpus striatum was interpenetrated by acephalocysts the size of peas, so that the olfactory nerve was compressed, and atrophied to such an extent that as it lay on the under surface of the anterior lobe it appeared to be no thicker than a thread. The thalamus was so distended with acephalocyst hydatids that it was pressed forwards and obliterated the tænia Tarini, (tænia semicircularis?) while the lateral choroid plexus appeared like a string of pearls pressed into the thalamus from the same cause. The left lateral ventricle contained serum, and the plexus numerous small millet-like cysts resembling false hydatids. The cerebellum was quite free from the entozoa, but in the corpora olivaria and corpora pyramidalia, besides echinococci there were seventeen distinct cysticerci. The corpora quadrigemina were distended with an elastic cyst containing serum and a few acephalocysts, so that their outline was destroyed, particularly posteriorly, from whence a knot of acephalocysts ran into the left crus cerebelli. The crura cerebri and pons were free from hydatids; and there were no traces of softening in the brain, but at the points examined, it presented an opal-like colour. No hydatids were found in the spinal cord except on the fascia dentata. Numerous groups of acephalocyst hydatids and a few cysticerci were scattered through the lungs, but most numerously in the left upper lobe. Fifteen cysticerci were found in the heart, principally in the septum and around the origin of the pulmonary artery. Acephalocysts, echinococci, and cysticerci were disseminated through the liver and spleen. Acephalocysts studded even the mesentery and the peritoneal coat of the gall-bladder in great numbers. The kidneys were healthy, but a few cysticerci were found in the muscles of the back.

Klencke observes upon this case, firstly, that it was remarkable what extensive changes had taken place in the brain—masses of hydatids irritating and displacing important organs—with comparatively little disturbance of the nervous system. This he explains by the gradual and imperceptible nature of the morbid process. Secondly, that in the brain and the serous membranes the acephalocyst hydatids predominated, while in the spleen and liver, the echinococci were most numerous. This he notices as confirmatory of some of his experiments subsequently detailed, and of an opinion he had formed that the liver was the primary seat of the hydatids, and their appearance in the brain secondary. Thirdly, although the liver and spleen were loaded with the entozoa, no signs of their existence there were observed during life.

*Hydatids in the spinal cord and nerves.* Every species of hydatid have their habitat in the spinal cord and nerves, and may cause a corresponding disorder of function. Klencke has often detected encysted acephalocysts, echinococci, and cysticerci in particular nerves, causing a neuroma hydatidosum, and inducing atrophy.

*Hydatids in the liver.* The liver as a highly vascular organ is often the seat of hydatids, for they are commonly to be found where the vascularity is greatest. They are never seen when the capillaries are few, a proof that their ova are carried into organs with the circulation. At the commencement, the hydatides spuriæ are situate between the microscopic aggregates of the hepatic cells. The primary cells near to false hydatids have yellow corn-like contents, while those of the hydatid contain only clear or yellowish fluid with from two to three blastidia. The acephalocysts are also situate at first between the primary hepatic cells, but they subsequently excite capillary congestion and effusion when a general cyst is formed. The cysticerci are generally found singly or in groups, and always near the blood-vessels. They appear to avoid the hepatic cells which are filled with a saffron-coloured matter, and our author has found that the bile behaves to them as an active poison.

Klencke states that these parasites get into the blood directly through the veins, particularly when the general cyst gives way after inflammation of its membrane. He further states that in many cases, he has found hydatids of all kinds swimming in the blood, either singly, or in groups of three or four. The cyst will open into a gall-duct, and then either the bile poisons the contained entozoa, or they pass along it into the bowels where they either fix again or are evacuated with the fæces. The cheesy matter found in the liver in connexion with an earthy or cartilaginous deposit as a sequel of hydatid inflammation is simply a compound of proteine and fat, and never contains the ova or young of the animal.

Klencke proceeds to describe hydatids as found in the spleen, lungs, ovaria, arteries, and Fallopian tubes, in the testicles, kidneys, muscles, &c. The cysticercus cellulosa is usually found in muscles. Its favorite habitat is in the heart, and in the muscles of the back, tongue, and thigh; and is most usually observed in pigs. We however leave these to notice the

*Hydatids found free in the blood.* The capillaries are often found connected with a little bag filled with hydatids, the true nature of which cannot be ascertained on account of their minuteness, and there is often an interruption or rupture of the capillaries at the point where they are found. This circumstance in addition to another, namely, that hydatids are principally found in tissues rich in blood-vessels, led Klencke to form the opinion that the animals entered the system by means of the circulation. The discovery made by our author that they propagate as other animals by ova, is not only favorable to this idea, but at once sets aside the opinions of Cruveilhier and others as to the spontaneous origin of hydatids.

The microscopic bodies noticed occasionally in fresh-drawn blood, and which are at least foreign to it, cannot be positively described as hydatid ova. The blood of a man who had acephalocyst hydatids in almost every organ, as well as in the cellular tissue, was examined by Klencke, and he found in it small cheese-like conglomerations of cells, not unlike those he had observed in connexion with specimens of echinococci. Now acephalocysts are simply masses of ova of the echinococcus in an advanced stage of development, as has been stated already. Klencke therefore infers that the microscopic objects observed were really the rudiments of acephalocysts. But he has actually seen other species of hydatids in the blood. He found a cysticercus in the blood of the vena cava superior of a female, and an immense number of acephalocysts and echinococci in the splenic and portal vein of a person who had the liver and spleen affected with those entozoa. Further examples of this kind are as follows. In a vessel of the pia mater, a group of acephalocysts; in a capillary vessel of the conjunctiva of the upper eyelid an unencysted, well-developed specimen of the cysticercus; acephalocysts lying unattached in the cavity of the heart; an isolated large acephalocyst attached to the free margin of an aortal valve; encysted echinococci in the varix of a varicose vein in the leg of an aged female; acephalocysts in the blood evacuated from a tumour on the elbow: these facts show, in our author's opinion, that hydatids do really enter the circulation, and may block up the capillaries. As to the mode of entrance, there can be no difficulty, for often the hydatid cyst is in direct connexion with the mouth of a blood-vessel, nor is it at all impossible that they may make their way from the intestines into the circulation. If the facts be true, as stated by our author, (and we know not why they should be doubted,) it cannot be denied that hydatids do enter the circulation, and that they should be considered as a true *contagium animatum*. Ova once in the circulation, we can readily infer with our author that they will be deposited most numerously in those organs where blood-vessels are most numerous, as the brain, liver, spleen, lungs; and that their first nidus will be a capillary too small in diameter for their transit. Here they will be developed and reproduced; here inflammation, exudation, and the formation of a cyst will take place; and from hence ova may find their way into the circulation, and be carried to other parts of the body.

*Origin of hydatids from without.* If there be no spontaneous generation of hydatids within the body, they must enter the blood from without, and consequently must have an external origin and existence. Man has no hydatid which is not found in lower animals; that they are not more numerously and frequently transferred by the flesh and milk of the latter to man is to be ascribed to the circumstance that the bile is an active poison to them. They are also passed with the urine, fæces, and saliva of animals. When Klencke discovered hydatids in mineral waters, and in the dung of an asparagus bed, he formed the opinion that hydatids existed in nature as other animals, and that they were to be regarded as the matter of a contagion. This opinion he has fully confirmed by his

*Experiments in Inoculation with the Ova of Hydatids. Hydatis spuria.* Two sucking whelps and two kittens were "handled" as follows; a fine trocar was introduced about half an inch below the navel into the abdominal cavity, and false hydatids introduced taken from a fresh human brain. The wound was carefully closed, and the young animals returned to their parent; after a quarter of a year they were examined (the wound having healed without any ill effects,) and in the two dogs and one of the cats a number of false hydatids were found around the cicatrix on the inner surface of the abdomen. In the other cat hydatids were found lower down close to the bladder.

A kid six days old, and a kitten, were the next subjects of experiment. False hydatids were taken from the infected animals just mentioned, and injected into the left femoral vein of the kid which was then restored to its parent for four months. The kitten had milk given to it poisoned with hydatid cells taken from the infected dogs. When the latter was dissected, no hydatids were discovered; the kid was however differently situated. When examined at the end of four months, two cysticerci fully developed were found between the skin and fascia of the right shoulder, contained in a cyst the size of a bean. In the upper part of the right lung, there was a cyst, four lines broad and nine lines long, in which together with exudation-cells and congestion-cells, there was a number of very small false hydatids. The surrounding structure had been changed by an inflammatory process. A sparrow was made to swallow lively hydatids from this source; in eight days it was opened, and a mass of hydatids found in the intestinal canal, amounting to a much greater number than those swallowed. A number of very small mother cells of the false hydatids were introduced into the orbital cavity of an old dog; in thirteen weeks they had multiplied so extremely as to alter the position of the globe of the eye.

A series of researches on other animals has led Klencke to the conclusion that inoculation with hydatid cells was only practicable with animals not too wide apart in the scale. Birds can rarely be inoculated from man, and amphibia never. But birds may be inoculated from inferior animals, and frogs from birds. He found that generally when the cells were injected into veins they stuck fast in the first organ they came to and were there developed. Thus when the splenic vein of a dog was made the medium of inoculation, the hydatid cyst was found in the liver. The carotid of an old goat was opened, and hydatid cells from the human brain introduced. The animal got well after the operation, and in a month was examined, when a series of hydatid cells were found in the corpus callosum, and in the optic nerves. Cells taken from the vesiculæ seminales of a man were injected into the uterus of a bitch. In nine months the post-mortem examination was made, and the organ was found full of hydatids. From this experiment Klencke very fairly infers that a female might be literally infected during coitus.

*Inoculation with acephalocysts and echinococci.* Klencke has found true acephalocysts in the milk of the cow. He found, by experiment, however, that the gastric fluid out of the body acted as a poison to the animal. Nine were given nevertheless to a cat in milk, and the animals were found in full reproductive activity, seated in the upper part of the ileo-colic valve. Several experiments are detailed analogous to those described in the last paragraph, which leave no doubt, if they be correctly stated, as to the facility with which these entozoa may be transferred from animal to animal. As the ova are not larger than from $\frac{1}{400}$ to $\frac{1}{800}$ of a line, they can readily pass along the smallest blood-vessels. And this explains why it has happened that when a part of the body has acquired a greater vascularity, as for example, after a blow on the eye, a hydatid has been developed. Klencke made some experiments on this point. He injected a fluid containing ova of echinococci into the veins of two dogs, two cats, and a guinea-pig. He wounded one dog in the tongue, another in the back, one cat he struck on the liver, and the other had the globe of the left eye squeezed. The guinea-pig was pinched on the thigh until it was black and blue. The guinea-pig and the cat with bruised liver showed hydatids when examined at the place of injury: in the others the experiment had no result.

Alcohol, iodine, bile, and urine, act as poison to these animals; but they suffer no change from antimony, arsenic, or mercury.

*Inoculation with polycephali.* The dimension of the reproductive germs of hydatids, as well as their relative fluidity, are of importance in determining their contagious power, because it is necessary that they enter the circulating system. Klencke made the following experiments to determine whether diffluent polycephali retained their vital power. He took hydatids of this kind, from the brain, placed them under a glass, and moistened them from time to time with a few drops of water. They dissolved into a slimy mass, which when mixed with serum became quite fluid. He inserted this fluid in the brain of a whelp and an old cat, in the mucous membrane of a he-goat, in the femoral veins of three young dogs, and he gave some of it in milk to two young cats. These experiments were performed in the presence of several friends. In eight weeks the animals were killed and inspected. In the brain of the whelp, at the wounded point, he found a cænurus (polycephalus) with a full-developed circlet and four germs. The old cat showed no traces of hydatids, neither did one of the cats to which poisoned milk was given, but in the left ventricle of the other he discerned a granular mucus, which was resolved by the microscope into cells and minute germs. In the he-goat, the right middle lobe of the brain contained a distinct polycephalocyst. Of the three dogs only one was affected. In this, a young cænurus with two cervical buds or germs was found in the fourth ventricle. Hot water is fatal to this species of hydatid.

*Inoculation with the cysticercus.* No hydatid is so easily communicable as this. Klencke has injected minute germs scarcely larger than blood-vesicles into the veins, and a brood of cysticerci in several organs of the body has been the result. Pork is often alive with them (*wimmelndvoll.*) The larvæ can make their way into the circulation by boring with their pointed head through the vessels. One experiment he relates is remarkably interesting. The blood of a sucking pig, newly killed, appearing, on a microscopic examination, to contain foreign particles, and having detected a cyst of cysticerci in the right ventricle of the animal, heart, as well as other parts of the body, he injected some of its blood into the veins of three cats. This was done in December, 1842, and two of the cats were sent to his friend, Director Helmuth, with a request that he would inspect them, which was done in the following May. In one which suffered from convulsive paroxysms resembling epilepsy, the convolutions of the brain were seen to be infected with cysticerci; in the upper part of the right lung of the other there was a solitary individual with a number of brood-cells. The third cat, kept by himself, pined away, so that it was necessary to examine it in February. An aneurismal dilatation of a branch of the pulmonary artery contained an unencysted group of cysticerci of various sizes, the largest of which contained ova.

*The therapeutics of hydatid formations.* Klencke found laurel-water to act as an active poison to the cysticercus. Other therapeutic agents possessing this property with regard to hydatids in general are camphor, ethereal oil of cubebs, acetic acid, balsam of copaiba, spirits of turpentine, ammonia, and the carbonates. Klencke found electricity to be a powerful destructive agent. He tried it clinically indeed on a man aged 54, who passed acephalocysts and echinococci with the urine. For this purpose he introduced one pole of a small galvanic battery through a glass catheter into the bladder; the other pole was sometimes applied externally to the abdomen, sometimes by means of a glass tube to the rectum, and sometimes to the lumbar region with the best results, dead hydatids being discharged about four hours after each application until they ceased altogether.

*Trichina spiralis.* A minute description of this parasite (now well known,) and several experiments are detailed elucidating its habits, and the facility with which it may be communicated from animal to animal. He denies that their habitation is in the voluntary muscles exclusively, as stated by Kobelt and Bischoff. He has observed them for the most part in men of hydropical or asthmatic constitution, but has never found that they interfered with the action of the muscles in which they were seated. The cyst containing them often appears to be an obstructed blood-vessel, as its fibres can be traced into a still pervious canal, and is liable to petrifactions—crystals of carbonate and lactate of lime, and even of silica, being deposited in it. This change is fatal to the worm, which also, when dead, becomes surrounded with earthy deposit, and leaves a hollow cast of itself in the latter.

This parasite remains lively after putrefaction has commenced in its residence: it is perfectly well when placed in fresh blood, and if dried up for several days, and then moistened with water, it is as vivacious as ever in a couple of hours. Klencke has no doubt that these parasites and their ova may and do exist out of the body. The ova are extremely minute, and can pass through very fine blood-vessels.

*Distoma hepaticum.* Klencke has ascertained that the structure usually considered as the ovum of this animal is, in fact, a cyst containing ova. These ova are cells so microscopically minute as to be able to pass along the smallest capillaries. The liver of the sheep is seldom free from them: only two were so in twenty-one that Klencke examined. They are also to be found in other organs, as the mammary glands, thymus, &c. It appears probable that the distoma passes through two or three stages of development, and that one of these stages at least is completed externally to a living organism—that, in fact, the animal returns to a living body to deposit its ova. The distoma may be dried without destroying its vitality.

*Intestinal worms.* The general results of Klencke's researches into the habits of this class of entozoa are as follows. They all produce ova, which are excreted with the fæces. These ova are developed externally to the body, taking up their abode either in their native excrement, or in ditches and pools of water. Being metamorphosed, they then return to the living organism, not by means of the alimentary canal, but through the blood, entering the circulation, and being deposited in various organs, from whence they make their way to the intestinal canal for the purpose of reproduction. The entozoon is guided to this point by the sexual instinct, as a suitable nidus for its ova, just as we find the œstrus and other insects selecting particular sites in animals whereon to deposit their ova.

Klencke has found entozoa in the blood of fishes, frogs, various mammals, and man, between which and animalculæ found in stagnant waters neither himself nor his friends could detect any difference whatever. These animals possess the faculty of passing through porous substances by means of their pointed head, or else bore through them with their spine-like tail. They are of various sizes, sometimes equalling in length the diameter of a blood-vesicle, sometimes one tenth of a line, a difference of size indicating a difference of genera or species. They all have an ovary, and the larger contain within them smaller animalculæ, varying in no respect (except in size) from themselves.

Various questions naturally arise in considering the natural history of intestinal worms. Are they and the animalculæ above referred to identical? Are those found in the blood developed into adult worms, and those found in stagnant water developed from the ova of these worms? The ovaries of the hematoidea (ascaris) contain about fifty millions of ova: what becomes of all these germs of animal life? How is it that only ova and adults are found in the intestines? To resolve these questions Klencke instituted a series of researches on—

*The development of the ova of the hematoidea (ascaris)*. Our author took a quantity of mucus which a child, aged 5 years, had evacuated per anum, and which he found to contain an immense number of ova of the ascaris, and he disposed of it thus: he mixed it with sand and rain-water in a small vessel, covered it over with a porous cover, and then placed it in a flower-pot filled with earth, leaving, however, the surface exposed. At five successive periods of from fourteen to twenty-one days, he examined the ova of five pots thus prepared, with the following results. The first showed the ova somewhat larger; in the second small embryos were seen coiled up: some of these were introduced into the peritoneal cavity of a whelp. The third pot was a failure, on account of its contents having dried up, from its too great porousness. In the fourth the mucus and ova had disappeared, and there appeared a great number of small worms from $\frac{1}{100}$ to $\frac{1}{40}$ of a line in length: they moved somewhat. An ovary could be detected in them, and they all had a sharp lancet-shaped tail, which they seemed to use as a borer. A fluid containing as many as possible of these animals was injected into the femoral veins of a kitten and a frog. The fifth and last pot was a blank—nothing was to be seen but the dry remains—the spolia opima of those in the fourth pot, their short, lancet-like tails were alone distinguishable. Klencke supposes that they died from being confined in the pot, and being thus prevented migrating to a locality suitable as regards food, &c. to their new stage of development,—just as tadpoles would have died under similar circumstances.

The whelp that was infected from the second pot showed no results; but the case was quite different with the frog infected from the fourth pot. To our author's great delight, three days after the operation he saw thread-like worms, measuring $\frac{1}{100}$ to $\frac{1}{180}$ of a line in length, circulating with the blood through the vessels of the web of the foot. He watched them by the hour, and could have no doubt whatever as to their identity with those he had previously injected into the femoral vein. He also found them in blood taken from the frog, and numerous cysts containing the animal were found in various organs—in the peritoneal cavity and beneath the peritoneum of the liver, spleen, in the large vessels, the substance of the heart, &c. Klencke argues that the nature of the frog, although not unpropitious to the life of the animal, was not so favorable to its proper development as the body of a mammal would have been. He therefore took a number of these thread-like worms from their cysts in the frog, and mixed them with fresh-drawn blood from a dog. Having ascertained that they were lively in their new medium, he injected the blood into the femoral vein of a dog, and in six weeks made a post-mortem examination of the animal. No traces of the worm were found in the blood, but a number of cysts, like those observed in the frog, were distinctly discernible beneath the peritoneal covering of the small intestines. Some were empty, but having a minute opening corresponding to the intestines, and the latter contained a number of ascarides about an inch in length. But what was most remarkable, a small thread-like worm was discerned beneath the mucous membrane of the intestine, its lancet-like tail bent into a curve.

Ascarides are remarkably tenacious of life. If they be dried, moisture revives them; or if only a portion of the dried worm be moistened, that portion will move. Even ascarides hardened in alcohol will revive when put into water. The ova of the bothryocephalus are equally as tenacious of vitality: pomegranate bark is poisonous to the adult, but has no effect on the ova.

There are two species of bothryocephalus, namely, B. latus and B. punctatus. The latter is unfrequent in the human subject, but common in the cottus scorpius. The B. latus has an ovary and vagina in each segment, and 700 testes, the connexion of which with a penis Klencke states he has in some instances been able to trace. Connected with these there are about 1600 glands, their ducts uniting into two common ducts, which are in immediate relation with the ovary. In the B. punctatus, the openings on one side are male, and on the other female. The animal is adherent to the appendices pyloricæ of the cottus, and just below the pylorus in man it undergoes changes according to the season, all the segments being detached from the head and thrown off at the commencement of winter, and each containing thousands of ova. New segments are formed, but the sexual organs are not developed in these until the spring. The segments are of course hermaphrodite and impregnate themselves, as in fact has been distinctly observed. Klencke very naturally inquires the fate of the multitudes of the ova excreted from man and animals. He attempted to perform the experiment with them, which he successfully practised with the ova of the ascarides, but without result. He gave the ova to dogs in food, and injected them into the veins. The worm was reproduced in the animal by the former means, but not by the latter.

The tænia solium is viviparous, that is to say, the ova are developed into small tæniæ within the ovary. These young microscopic animals can live out of the body. Klencke kept some in water alive for fourteen days; he then dried them, and restored them to life by moistening them. He has accidentally discovered them in ditch water, and a friend assured Klencke that he had detected the same animal in the water of Vienna. The tænia ought therefore to be endemic in that city; and this appears to be the case. (Vide our review of Dr. Wawruch's work on the Tænia, vol. XVIII, p. 324.) Klencke gave some of the small tæniæ, which he dried and brought to life again, to a cat and a lamb; in both adult entozoa were developed. The microscopic tæniæ have a lancet-shaped head adapted to the duty of piercing animal membranes, and he supposes it is by the use of this that they get into the intestines of the human fœtus.

The natural history of the tricocephalus and oxyuris vermicularis has not as yet been investigated by our author, but he remarks that he has detected microscopic examples of the tricocephalus dispar in the ditch water of a moor, and thinks it probable that like ascarides and tæniæ, this animal also passes through a stage of development externally to the organism.

We have now placed a full and fair account of Klencke's views before our readers, and although we cannot but think that many of his experiments are inconclusive, it is clear that the general results at which he has arrived warrant us in recommending them to the notice of practised microscopists.

